# The effect of temperature on the evolution of per offspring investment in a globally distributed family of marine invertebrates (Crustacea: Decapoda: Lithodidae)

**DOI:** 10.1007/s00227-015-2776-8

**Published:** 2016-02-12

**Authors:** Sven Thatje, Sally Hall

**Affiliations:** Ocean and Earth Science, University of Southampton, National Oceanography Centre, Southampton, European Way, Southampton, SO14 3ZH UK

## Abstract

Within the marine environment, per offspring investment (POI) is associated with modes in larval development; an increase in POI has often been described with a decrease in temperature, as evidenced along latitudinal clines. However, the environmental drivers of POI remain largely hypothetical and have not yet been tested within an evolutionary context. Here, we test the hypothesis that developmental temperature is linked to POI within a globally distributed and diverse family of benthic crustaceans, the Lithodidae, also known as stone or king crab. To do this, we examine variations in egg diameter—a proven corollary of POI—within the Lithodidae. Based on a rare case of well-construed phylogeny, we test the relationship between egg diameter and two aspects of the maternal physical environment: water depth and temperature. We observe a significant relationship between decreasing environmental temperature and an increase in POI within genera of lithodid crabs, and independent of depth. There is a clear correlation of high levels in POI with a decrease in temperature in lithodid crab genera currently inhabiting the deep sea, all of which follow a food-independent (lecithotrophic) mode of larval development. In contrast, lithodid genera thriving in the warmer waters of shallow (continental shelf) seas follow a feeding (planktotrophic) mode in larval development. We conclude that temperature is an important factor governing POI, and discuss its importance in the evolution of larval lecithotrophy in marine invertebrates.

**Electronic supplementary material:**

The online version of this article (doi:10.1007/s00227-015-2776-8) contains supplementary material, which is available to authorized users.

## Introduction

### The role of egg size in invertebrate reproduction

The amount of energy invested into offspring by the mother has a direct impact on the length of larval development, resistance to starvation in larvae and the size of larvae at hatching and metamorphosis (Sinervo and McEdward 1988; Reitzel et al. 2005; Oliphant et al. 2013; Oliphant and Thatje 2013). An easy-to-obtain ecological parameter, egg size, has frequently been used as a corollary of per offspring (maternal) investment (POI; Jaeckle 1995; Anger 2001; Moran and McAlister 2009). Per offspring investment, in most cases reflected in egg size, dictates whether larvae of aquatic invertebrates develop into planktotrophic (feeding) or lecithotrophic (non-feeding) stages (Miles and Clark 2002). The hypothesis that egg size is proportional to energy content (e.g. Oliphant et al. 2013; Oliphant and Thatje 2013) has been supported by cases in which empirical assessments between species were made (Clarke 1993; Jaeckle 1995; Anger et al. 2002; McEdward and Morgan 2001). In this context, several physical factors have been investigated for their correlation with egg size in an attempt to resolve the various effects of the environment on POI. Water temperature, salinity, water pollution, maternal size and condition at reproduction all seem to play a part in the partitioning of energy into eggs, although trends are not necessarily consistent across phyla (Clarke 1987; Atkinson 1994; Bernardo 1996; Wehrtmann and Kattner 1998; Collin 2003; Fischer et al. 2009a, b). Stochastic variability of egg size has also been examined, and the influence on interspecific variability was found to be insignificant (McEdward and Morgan 2001).

Temperature-driven latitudinal shifts in energy provisioning into eggs have long been suggested for some invertebrate orders, especially decapod crustaceans and molluscs (Thorson 1950; Hart and McLaren 1978; Clarke 1987; Ernsting and Isaaks 1997; Arntz and Gili 2001). A trend in an increase in egg volume with water depth has also been described in neogastropod molluscs (Buccinidae) and decapod crustaceans (Munidopsidae, Chirostylidae) (Smith et al. 2015). Furthermore, a study of latitudinally widespread cancrid crab showed that—in the absence of food limitation—a shift in embryonic nutrition from protein to lipid metabolism is entirely dependant on temperature and on the lower temperature range tolerated by the species. This is reflected by a latitudinal cline in egg, as well as larval size and morphology (Thatje and Bacardit 2000; Fischer et al. 2009a, b; Weiss et al. 2010). The scarce evidence for a link between reproductive ecology and temperature along latitudinal (temperature) clines has often been attributed to increased seasonality rather than temperature alone (Clarke 1987; Thatje et al. 2003). Recent work focusing on the intraspecific level suggested that temperature is the main driver of energy allocation into offspring (Giménez 2006; Oliphant et al. 2013; Oliphant and Thatje 2013; Gonzalez-Ortegón and Giménez 2014).

Despite the summarised evidence, the widely varying life histories and ecological traits that exist across marine invertebrate taxa have presented scientists with difficulties when trying to understand better the environmental factors that govern per offspring investment (e.g. Miner et al. 2005; Thatje et al. 2015). Large comparative works—to date—focus mainly on echinoderms and molluscs (Hadfield and Switzer-Dunlap 1984; Emlet et al. 1985; Lessios 1990; Atkinson et al. 2001; Collin 2003). Only one study on an anomuran crab species, the mole crab *Emerita analoga*, demonstrated a lack of correlation between egg size and temperature on beaches in California (Dugan et al. 1991). To the best of our knowledge, there is no study available that describes the drivers of POI and within a well-construed phylogenetic scenario (but for discussion see, Strathmann 1985).

### Evolutionary history and biogeography of the Lithodidae

The family Lithodidae comprises two subfamilies: the Lithodinae and the Hapalogastrinae. The Hapalogastrinae are small, shallow- or intertidal-dwelling crabs with soft (uncalcified) abdomens. Their range is restricted to the high latitudes of the north Pacific, where temperatures are below 16 °C during the period of larval development (Hall and Thatje 2009). The Lithodinae have a wide diversity of ecological types, with six intertidal or subtidal genera in the north Pacific, and four genera with wider bathymetric ranges inhabiting cold waters (predominantly in the deep sea) globally (Zaklan 2002; Hall and Thatje 2009).

**Fig. 1:**
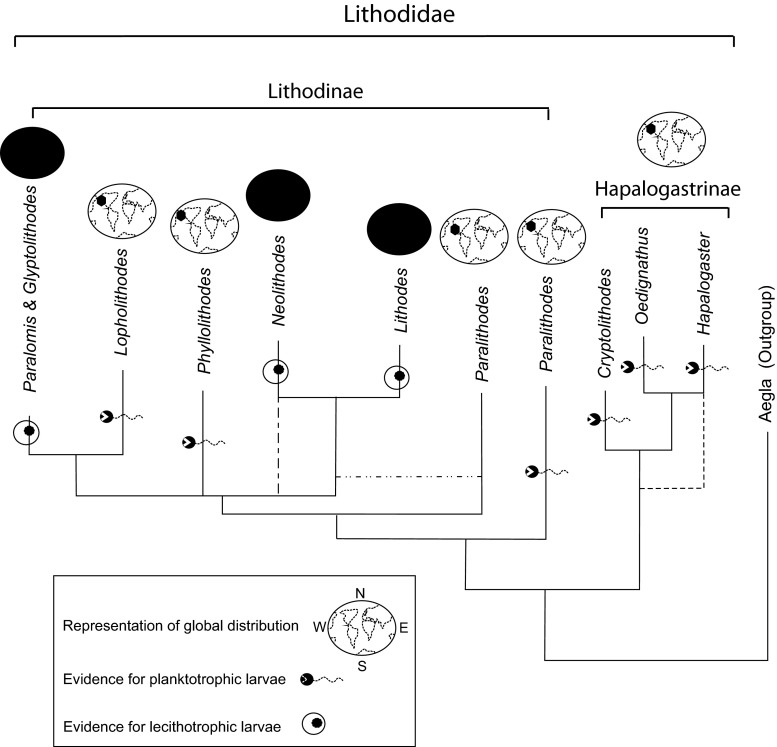
Schematic phylogeny of the Lithodinae and Hapalogastrinae adapted from Hall (2010). Representation is based on Bayesian analyses of an alignment of 39 lithodid species belonging to 10 genera using genes COI, 16S, 28S and ITS1. Species within monophyletic genera are condensed to a single taxon label, and polyphyletic or paraphyletic genera are indicated as such by multiple taxon labels (*Paralithodes*). Less frequent alternative topologies are indicated by *dotted lines* on the same tree. Probabilities based on Bayesian analysis represented at internal nodes. Outgroup species are taken from the anomuran genus *Aegla* (species *platensis*, *intercalata*, *longirostris*; see Hall 2010, for further details). Maps indicate the distribution for each taxonomic lineage (global = *dark circle* vs. north Pacific); larval developmental type is indicated as planktotrophic (feeding) larvae or lecithotrophic (non-feeding) larvae

Molecular and distributional evidence indicate that lithodids evolved from ancestors inhabiting kelp holdfasts in the shallow waters of the north Pacific (Cunningham et al. 1992; Zaklan 2002), and it is most parsimonious to assume that such ancestors had a planktotrophic larval phase (Fig. 1; lithodid phylogeny after Hall 2010). Lithodid groups that submerged and radiated into deep waters outside the north Pacific are thought to have made the transition from ancestral larval planktotrophy to lecithotrophy (Cunningham et al. 1992; Hall and Thatje 2009). “Deep-sea” forms of the Lithodidae are known to have emerged a number of times into shallow waters in subantarctic South America (3 cases: *Lithodes santolla*, *L. confundens* and *Paralomis granulosa*) and subarctic north Atlantic (1 case: *L. maja*). In these cases, shallow-water crabs share a lecithotrophic mode of larval development (for discussion see, Strathmann 1985) with their deep-sea relatives (Anger 1996; Thatje and Mestre 2010).

Experimental data have shown that many species of Lithodidae have fully food-independent (lecithotrophic) larval development (Anger 1996; Calcagno et al. 2004; Morley et al. 2006; Watts et al. 2006; Thatje and Mestre 2010; Shirley and Zhou 1997; Calcagno et al. 2003). An obligatory non-feeding larval mode is accompanied by physiological and anatomical traits in lithodids, such as the reduced development of larval mouthparts (Campodónico and Guzmán 1981; McLaughlin et al. 2001; Watts et al. 2006) or a lack of digestive enzymes (Kattner et al. 2003; Saborowski et al. 2006). In contrast, lithodid species of the genus *Paralithodes* (Paul et al. 1989; Eppelbaum and Borisov 2006) as well as any species of the Hapalogastrinae studied to date (Crain 1999; Kim and Hong 2000; Duguid and Page 2009; Jensen 1995; Hong et al. 2005) are known to have food-dependent zoeal stages. Zoeal planktotrophy is found in north Pacific Lithodidae but is not known in any lithodid from outside that region.

This paper examines egg traits indicative of per offspring investment (POI) and larval developmental mode within the globally distributed decapod crustacean family Lithodidae. For the first time, we link physiological and ecological traits to the global radiation of this large family of mobile benthic crustaceans (after Hall and Thatje 2009). Temperature has long been proposed to drive POI within latitudinal clines (generally known as “Thorson’s rule”); however, there is currently no study available that attempts to test this macroecological observation in an evolutionary context.

## Materials and methods

### Acquiring egg data

Lithodid egg samples were obtained from the National Museum of Natural History, Smithsonian Institute, Washington, USA, the Muséum national d’Histoire naturelle, Paris, France, the Natural History Museum, London, UK, the Centro de Estudios Avanzados de Blanes, Spain, and the Senckenberg Forschungsinstitut und Naturmuseum, Frankfurt, Germany. Egg data used in this study were obtained from females stored in 70 % ethanol and compared with data obtained from the literature. We collected original egg-size data from ovigerous females (38 species, one female each; 9 lithodid genera; Supplementary Information). Eggs held on the fifth abdominal pleopod were removed by scalpel and placed into a vial of 70 % ethanol for later analysis. Egg measurements, accurate to the nearest 0.02 mm, were taken using an eyepiece graticule on a Leica dissecting microscope. Only eggs that were intact and not deformed were used for measurement. Developmental stage of the embryo was considered in analyses, and eggs were classed as unfertilised/early, uneyed or eyed. Only early development eggs, which are of spherical shape, were considered in this study; no samples were considered that contained late-stage (with developing or fully developed eyes visible) embryos. In all cases, the length of the egg was measured. For this study, we randomly selected and measured at least 30 eggs from each individual.

### Preservation analysis

**Fig. 2:**
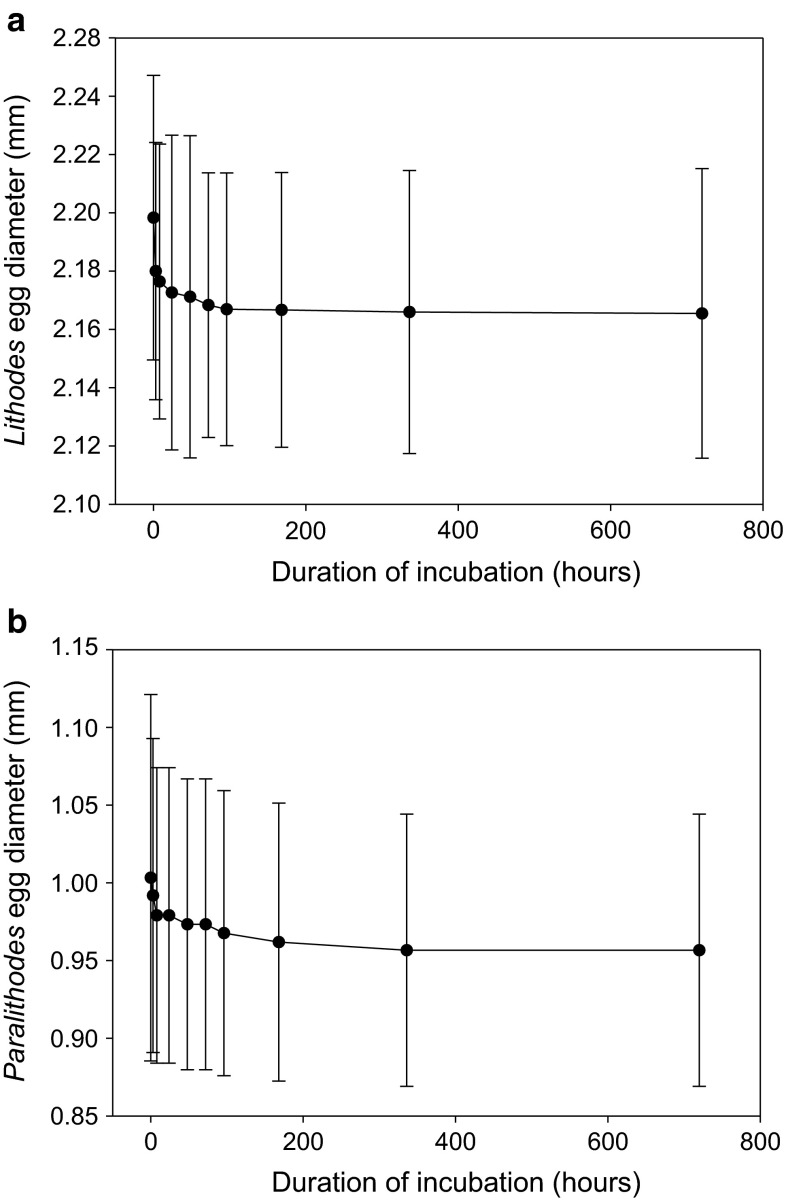
Effect of ethanol preservation on egg size. Range in diameter of eggs taken from *Lithodes confundens* (**a**) and *Paralithodes camtschaticus* (**b**) after a period of incubation in 70 % ethanol. Eggs are removed from the fifth pereiopod of a fresh ovigerous female. Measurements of 30 eggs are taken using an eyepiece graticule at each time interval shown

An initial comparison of our data with that from the literature indicates a slight effect of ethanol preservation on egg size (Fig. 2). To test for the effect of methodological bias related to preservation, we compared the diameters of 30 randomly selected fresh eggs with those preserved for up to 1 month in 70 % ethanol. For this, we selected one species with lecithotrophic (non-feeding) larval development (*Lithodes confundens*, Fig. 2a) and one with planktotrophic (feeding) development (*Paralithodes camtschaticus*, Fig. 2b).

### Assessment of in situ temperature

**Fig. 3:**
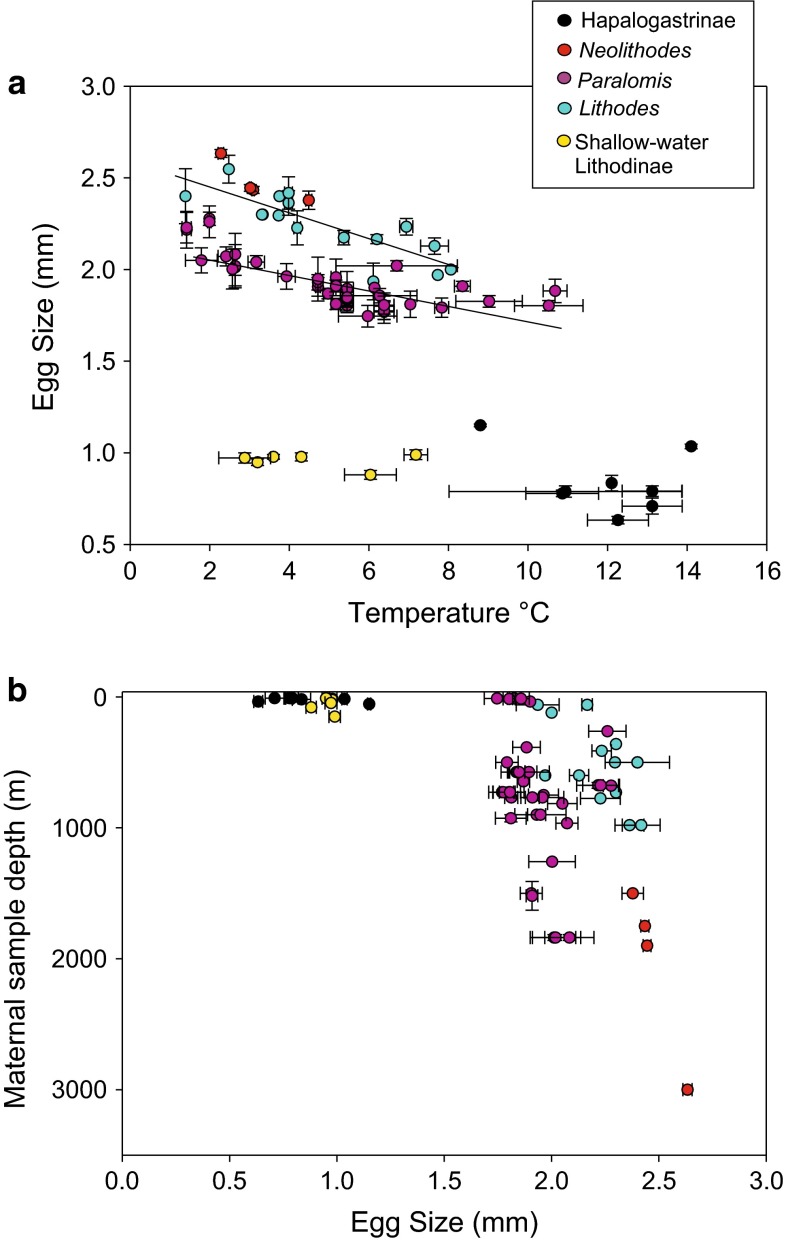
Effect of depth and temperature on egg size in the family Lithodidae. Average size of eggs taken from individual lithodids, classified according to genus. Figured in relation to **a** environmental temperature of the location of catch for the ovigerous females from which eggs are sampled. Temperature data extracted from the World Ocean Atlas (cf “Materials and methods”) using sample coordinates and month of capture to obtain estimates of temperature at location (*horizontal error bars* depict the range of temperatures recorded at each station, if known; vertical error bars depict range in egg diameter for each individual sampled). *Regression lines* are shown for genera *Paralomis* and *Lithodes*; **b** depth of sampling of the ovigerous females from which the eggs are obtained; horizontal error bars depict range in egg size for each individual sampled (for details, see “Materials and methods”) [Groups represented in this figure: *Neolithodes*: 2 species; *Lithodes*: 10 species; *Paralomis*: 17 species; Shallow-water Lithodinae includes genera (*Paralithodes*, *Rhinolithodes*, *Lopholithodes*): 4 species; Hapalogastrinae includes genera (*Cryptolithodes*, *Oedignathus,* *Hapalogaster*): 5 species]

In situ water temperature at time of sampling was obtained revising sampling location data available from museum collection archives and for each ovigerous female used in the present study. Where a local in situ temperature was not recorded at the time of capture, the temperature was estimated from data in the World Ocean Atlas (Locarnini et al. 2006) using latitude, depth, longitude and month of recovery from sampling records. In addition, variation in temperature at sampling locations (if known) has been visualised (Fig. 3).

### Per offspring investment and phylogenetic relatedness

We assessed the distribution of egg traits for different lineages of the Lithodidae (subfamilies: Lithodinae and Hapalogastrinae) and based on established phylogenetic reconstruction based on Bayesian analyses of an alignment of 39 lithodid species belonging to 10 genera, using genes COI, 16S, 28S and ITS1. Species within monophyletic genera are condensed to a single taxon label; polyphyletic or paraphyletic genera are indicated as such by multiple taxon labels (for further details, see; Hall and Thatje 2009; Hall 2010).

In the Lithodidae, eggs size <1.5 mm is known to represent a planktotrophic (feeding) mode in larval development, representative of shallow-water genera of the Hapalogastrinae (*Oedignathus, Hapalogaster, Cryptolithodes*), as well as for the shallow-water genera of the Lithodinae (*Paralithodes, Lopholithodes, Phyllolithodes*, Fig. 2). The three most speciose lithodinid genera (*Lithodes*, *Neolithodes*, *Paralomis*) as well as *Glyptolithodes* have eggs >1.5 mm in size and are considered to follow a lecithotrophic (non-feeding) mode in larval development (Fig. 1); most of these inhabit the deep sea. The early ontogeny of the Lithodidae remains one of the best-studied cases for divergent reproductive traits in the early development of marine invertebrates; evidence for developmental modes and larval energetics are largely based on an extensive body of the literature, which provides the baseline to this study (for review see; Thatje et al. 2005; Hall 2010; Thatje and Mestre 2010).

### Statistical analyses

We applied the Holm–Sidak method of pair-wise comparison for normally distributed data, the average size of eggs produced by individuals in the genera of the Lithodidae. We used this test to correct for multiple *t* test and in order to present significance between genera; the Sidak modification of the Holm test makes it a more powerful, especially when applied to many comparisons at once (Šidák 1967; Holm 1979). Furthermore, we assessed egg size variability with regard to temperature and depth using polynomial regression analysis. For two genera with the largest available datasets, *Lithodes* and *Paralomis*, statistical polynomial regressions were calculated and in order to test for a unimodal relationship with a single maximum or minimum value.

## Results

### Egg preservation analysis

In a comparison of ethanol-preserved and fresh eggs, each batch of preserved eggs lost 1.5 % in *Lithodes confundens* and 4.5 % in *Paralithodes camtschaticus* of their diameter over the course of a month compared with the mean measurement of unpreserved eggs (Fig. 2). In both species, most of the change in egg size occurred within the first 12 h of preservation, with an insignificant decrease in egg size from 24 h onward. All of the data used in the main study were taken from eggs preserved for more than 24 h in 70 % ethanol, and this slight skew is accepted as a uniform bias in the data which should not affect the interpretation of the results.

### Egg size variability within genera

Genera that are predominantly found in the deep sea—*Neolithodes*, *Lithodes* and *Paralomis* (including shallow-water species *P. granulosa*, *L. santolla*, *L. confundens*, *L. maja*)—have eggs more than 1.7 mm diameter (Fig. 3a, b; also see; Supplementary Material). Using the Holm–Sidak method of pair-wise comparison for normally distributed data, the average size of eggs produced by individuals in genera *Lithodes* (2.24 mm) and *Neolithodes* (2.47 mm) was found to be significantly higher (*p* < 0.01) than those in members of the genus *Paralomis* (1.93 mm).

Shallow-water representatives of the Hapalogastrinae (*Oedignathus, Hapalogaster*, *Cryptolithodes*) and Lithodinae (*Paralithodes*, *Lopholithodes*) have average egg diameters ranging from 0.63 to 1.15 mm and are significantly smaller (*p* < 0.01) than those produced by deep-sea genera (*Neolithodes*, *Lithodes* and *Paralomis*).

### Egg size variability with regard to temperature and depth

Within the members of deep-sea lithodid genera (*Neolithodes*, *Paralomis, Lithodes*), including species that currently inhabit shallow waters (see previous section) egg size decreases with increasing temperature (Fig. 3a). The largest eggs (some >2.4 mm) are observed in the abyssal genus *Neolithodes*, which inhabits waters up to 3500 m deep where temperatures are between 1 and 4 °C.

For two genera with the largest available datasets, *Lithodes* and *Paralomis*, statistical regressions were calculated. Linear regression indicated a relationship between egg size and environmental temperature (*r*^2^ = 0.68; intercept = 2.588, slope = −0.0726) for genus *Lithodes* and (*r*^2^ = 0.569; intercept = 2.15, slope = −0.046) for genus *Paralomis*. Within the genus *Paralomis*, a further second-order test of regression demonstrated a strong relationship (*r*^2^ = 0.81) between egg size and a function of environmental temperature.

No strong relationship was found between egg size and sample depth (a correlate of hydrostatic pressure) for the genera *Paralomis* and *Lithodes* (*r*^2^ = 0.0703 and *r*^2^ = 0.338, respectively; *p* values <0.01).

### Per offspring investment and phylogenetic relatedness

Reproductive traits in the Lithodinae are strongly lineage specific, with the deep-sea genera *Lithodes*, *Paralomis* and *Neolithodes* demonstrating a lecithotrophic mode (egg size >1.5 mm) in larval development, whereas the shallow-water genera *Lopholithodes*, *Phyllolithodes* and *Paralithodes* have a planktotrophic mode (<1.5 mm) in development. The shallow-water Hapalogastrinae (genera; *Cryptolithodes*, *Oedignathus* and *Hapalogaster*) also follow a planktotrophic mode (egg size < 1.15 mm) in larval development (see Fig. 1; phylogeny after Hall and Thatje 2009). There are few species of the Lithodinae occurring in shallow seas (*P. granulosa*, *L. santolla*, *L. confundens*, *L. maja*), all of which also show lecithotrophy in larval development (Campodónico and Guzmán 1981; Anger 1996; Kattner et al. 2003; Calcagno et al. 2004).

## Discussion

### Methodological considerations

This study is making use of lithodid crab egg samples obtained from various international museum collections. We experimentally assessed the potential effect of (ethanol) preservation of eggs in order to consider any potential bias of long-term storage on egg size. We found a minor, but consistent effect of preservation on the size of eggs. Eggs shrink by 1.5–4.5 % of their original size after preservation in 70 % ethanol (Fig. 2). However, this is not expected to affect the analysis, since all specimens had been preserved in 70 % ethanol (also see: Zaklan 2002; Hall 2010).

In this analysis, we used in situ temperature at each sampling location and time of year as a parameter for assessing temperature-driven effects on per offspring investment, as indicated by egg size (Anger et al. 2002). Seasonal bathymetric migration into shallower—and warmer—waters is known in a few species of shallow-water living (continental shelf) Lithodinae and most likely for the purpose of larval release. Such migration into warmer waters may potentially affect the use of energy by embryos and reduce egg size (e.g. *Paralithodes camtchaticus*, Jørgensen and Nilssen 2011; *Lithodes confundens*, Lovrich et al. 2002). However, our egg data refer to the early stage in embryo development (see “Materials and methods”), which is taking place in deeper waters. In addition, oogenesis—which is affecting POI—is also taking place in deeper, cooler waters, justifying the present method. Nevertheless, any such potential thermal effect of increased temperature in shallower waters would, indeed, lead to an underestimation of the already significant effect of temperature on POI demonstrated in this study.

### Environmental influences on reproductive traits

There is a strong correlation between egg size and environmental temperature within lithodid genera *Lithodes* and *Paralomis*, and regardless of habitat depth. Variations in POI have previously been linked with other environmental factors including maternal diet or food availability, quality of oviposition site, density of conspecifics and predation risk (Fox and Czesak 2000; Anger 2001; Oliphant et al. 2013; Oliphant and Thatje 2013). Differences in offspring size within species are also commonly observed between seasons and along latitudinal or bathymetric gradients, and have been described by temperature effects (Clarke 1987; Wehrtmann and Kattner 1998; Morley et al. 2006; Rosa et al. 2007).

Temperature is likely to be a key mediator of phenotypic variation in all ectotherms (Atkinson 1994; Oliphant et al. 2013; Oliphant and Thatje 2013). Low metabolic rate and protracted growth of females at low temperature reduces the cost of somatic maintenance, increasing the proportion of available resources that can be allocated to vitellogenesis (Sheader 1996). At low temperatures, selection may also favour the production of larger offspring and promote the abbreviated development of lecithotrophic larval instars, as observed in cold-water adapted lithodid crabs from subantarctic and Antarctic waters (Campodónico and Guzmán 1981; Lovrich et al. 2003; Watts et al. 2006; Thatje and Mestre 2010). Such early life history adaptations to low temperatures have often been discussed to support larval survival in regions where the period of primary productivity is short or pronounced, such as at high latitudes or in the deep sea (Clarke 1993, Clarke 1987; Thatje et al. 2003).

Additionally, oxygen limitation of large embryos is more likely to occur at higher than lower temperatures (Davis 1975); in warm-water conditions, smaller eggs may be favoured for their lower surface/volume ratio, thus constraining egg size. Interestingly, recent work by Smith et al. (2015) for the first time indicated an increase in metabolic demand for larval development at lower temperature and under hyperbaric conditions, as representative of the deep sea. Such increase in metabolic cost observed under hyperbaric conditions may, therefore, contribute further to the long-standing hypothesis of an increase in POI at low temperatures, and by increasing the cost of reproduction (for discussion see, Oliphant et al. 2013; Oliphant and Thatje 2013). The present work does not provide any evidence for a depth-related effect on POI.

The factors governing the development of larger eggs are diverse and with temperature most likely being a key factor in driving POI in the Lithodidae. Indeed, the evolutionary age—or history—of a group of species or clade may be key in determining the role of an environmental factor plays in the selection for reproductive traits.

### Evolutionary drivers of lecithotrophy

Here, we have shown that significantly larger eggs are produced in colder waters within lithodid genera, indicating some level of physiological plasticity in POI in response to environmental conditions. Based on these data, we suggest environmental temperature to influence egg size (POI) and, by extension, larval developmental traits within lithodid crab lineages (Fig. 1). The effect of temperature on egg size as a correlate of POI, however, is of a much lower order than the size difference between eggs from shallow-water north Pacific lithodids and eggs from deep-sea lithodid genera. This profound division is further evidence for the divergent lithodid reproductive strategies between deep-sea and shallow-water lineages that have been shown in previous physiological experiments (see Introduction); therefore, the development of lecithotrophic (food-independent) larval developments in lithodid crabs has likely played a pivotal role in the radiation and diversification of the family. The evolution of lecithotrophy has previously been regarded a main—if not key—condition that allowed the three most diverse extant genera (*Lithodes*, *Neolithodes* and *Paralomis*) to radiate globally through the cold stenothermal environment of the deep sea, where the lack of continuous food supply selects against feeding larvae (Hall and Thatje 2009). The theory of linked submergence (migration to greater depth) and range expansion in deep-sea lithodid lineages is supported by genetic and other physiological (temperature, hydrostatic pressure) bottlenecks previously discussed for the family Lithodidae (Roughgarden 1989; McKitrick 1993; McEdward 1997; Hall and Thatje 2009; Hall 2010; Hall and Thatje 2011). In those lithodid genera with an expanded bathymetric range, the selection for lecithotrophy allowed larval development to take place over an extended period (lasting a year or longer), disconnected from limited periods of phytoplankton blooms in surface waters (Thatje et al. 2003, 2005; Thatje and Mestre 2010). This stands in strong contrast with most other lithodid genera that restrict their distribution to shallow waters of the north Pacific and have planktotrophic larval developmental stages (Duguid and Page 2009; Hall and Thatje 2009). Food limitation has frequently been discussed to be a main driver of lecithotrophic larval development in freshwater crabs and, undoubtedly, is likely to play an important role in the evolution of reproductive traits in marine invertebrates, too (for discussion see, Anger 1995).

Our finding that lower habitat temperature is a strong predictor of increasing egg size supports previous evidence that latitudinal temperature clines influence POI among other crustaceans and marine invertebrates (Thorson 1950; Clarke 1987; Wehrtmann and Kattner 1998; Fischer et al. 2009a, b; Weiss et al. 2010; Oliphant et al. 2013; Oliphant and Thatje 2013). Here, we advocate that temperature, and within a framework of previously established phylogeny, is the predominant factor influencing per offspring investment (POI). It is likely that the evolutionary history (“experience”) of a species affects the role of an environmental factor plays in the selection for species-specific reproductive traits.

## Electronic supplementary material

Below is the link to the electronic supplementary material.

Supplementary material 1 (DOC 92 kb)
