# Supplementary material for: The effect of temperature on the evolution of per offspring investment in a globally distributed family of marine invertebrates (Crustacea: Decapoda: Lithodidae)
Source: Mar Biol. 2016 Feb 12;163:48. doi: 10.1007/s00227-015-2776-8 (PMC4751184; doi:10.1007/s00227-015-2776-8)
Supplement: Supplementary file 1 — Supplementary material 1 (DOC 92 kb) [file 227_2015_2776_MOESM1_ESM.doc]

**Supplementary Information**

*Marine Biology* (Springer)

**The effect of temperature on the evolution of per offspring investment in a globally distributed family of marine invertebrates (Crustacea: Decapoda: Lithodidae)**

Sven Thatje* and Sally Hall

Ocean and Earth Science, University of Southampton, National Oceanography Centre, Southampton, European Way, Southampton, SO14 3ZH, UK

*Corresponding author. Email: [svth@noc.soton.ac.uk](mailto:smh57@noc.soton.ac.uk)

Tel: (+44) 2380 596449

**Data: Egg measurements**

**(one female per species, n=30 eggs; eggs chosen were in early stage of development = eggs spherical; embryos without visible eyes).**

From Direct Measurement of samples from collections

| ***Species*** | **Mean egg diameter** | **Range +/-** | **S.D.** |
| --- | --- | --- | --- |
| *Paralithodes brevipes* | 0.972222 | 0.28 | 0.055606 |
| *Paralithodes camtschaticus* | 0.8533 | 0.35 | 0.075668 |
| *Paralithodes californiensis* | 1.860575 | 0.32 | 0.096933 |
| *Paralithodes rathbuni* | 1.668 | 0.32 | 0.099679 |
| *Paralomis africana* | 1.834521 | 0.316 | 0.078411 |
| *Paralomis aculeata* | 2.076231 | 0.4 | 0.12671 |
| *Paralomis anamerae* | 2.276429 | 0.48 | 0.131081 |
| *Paralomis cristata* | 2.36 | 0.44 | 0.141825 |
| *Paralomis cristulata* | 1.883333 | 0.4 | 0.130537 |
| *Paralomis formosa* | 2.047778 | 0.48 | 0.135497 |
| *Paralomis elongata* | 1.95 | 0.425 | 0.154536 |
| *Paralomis granulosa* | 1.788043 | 0.30125 | 0.083936 |
| *Paralomis grossmani* | 1.861818 | 0.28 | 0.098518 |
| *Paralomis inca* | 1.806154 | 0.44 | 0.100272 |
| *Paralomis mendagnai* | 1.882222 | 0.32 | 0.04376 |
| *Lithodes tropicalis* | 2.363636 | 0.325 | 0.094366 |
| *Lithodes turritus* | 2.13125 | 0.3 | 0.116425 |
| *Lithodes mendagnai* | 2.252 | 0.58 | 0.138071 |
| *Cryptolithodes sitchensis* | 0.802667 | 0.18 | 0.05359 |
| *Hapalogaster cavicauda* | 0.633 | 0.16 | 0.040878 |
| *Rhinolithodes wossnessenskii* | 0.907692 | 0.16 | 0.048063 |
| *Lopholithodes foraminatus* | 0.989524 | 0.24 | 0.053051 |
| *Neolithodes asperimus* | 2.36 | 0.13 | 0.097678 |
| *Neolithodes capensis* | 2.63 | 0.1 | 0.040394 |

Egg measurements obtained from the Literature

| ***Species*** | **Mean Egg Size** | **Range -** | **Range**  **+** | **Source** |
| --- | --- | --- | --- | --- |
| *Cryptolithodes sitchensis* | 1.035 | 1.01 | 1.06 | Zaklan 2002 |
| *Cryptolithodes typicus* | 0.835 | 0.75 | 0.92 | Hart 1965 |
| *Lithodes aequispinus* | 2.295 | - | - | Hiramoto and Sato 1970 |
| *Lithodes aequispinus* | 2.4 | - | - | Jewett et al. 1985 |
| *Lithodes aequispinus* | 2.3 | 2.07 | 2.52 | Zaklan 2002 |
| *Lithodes couesi* | 2.3 | - | - | Somerton 1985 |
| *Lithodes ferox* | 1.97 | - | - | Abello and Macpherson 1992 |
| *Lithodes maja* | 2 | - | - | MacDonald et al. 1957 |
| *Lithodes murrayi* | 2.4 | 1.92 | 2.88 | Arnaud and Do-Chi 1977 |
| *Lithodes santolla* | 1.935 | 1.4 | 2.47 | Guzman and Campodonico 1972 |
| *Lithodes santolla* | 2.1 | - | - | Vinuesa 1987 |
| *Lithodes turkayi* | 1.7 | - | - | Lovrich and Vinuesa 1999 |
| *Paralithodes californiensis* | 1.75 | 1.62 | 1.96 | Zaklan 2002, K. Rypien pers.obs. |
| *Paralithodes camtschaticus* | 0.78 | 0.71 | 0.82 | Marukawa 1933 |
| *Paralithodes camtschaticus* | 0.95 | 0.88 | 1.03 | Matsuura and Takeshita 1985 |
| *Paralithodes platypus* | 1.18 | 0.98 | 0.98 | Saskawa 1975 |
| *Paralithodes platypus* | 1.2x 1 | - | - | Somerton and Makintosh 1985 |
| *Paralomis granulosa* | 2.1 | - | - | Vinuesa 1987 |
| *Paralomis granulosa* | 1.9 | - | - | Lovrich and Vinuesa 1993 |
| *Paralomis longipes* | 2 | - | - | Faxon 1893 |
| *Paralomis seagranti* | 2.5 | - | - | Eldredge 1976 |
| *Paralomis spinosissima* | 2 | - | - | Otto 1993 |
| *Rhinolithodes wosnessenskii* | 1.125 | 1.07 | 1.18 | Zaklan 2002 |
| *Hapalogaster cavicauda* | 0.775 | 0.71 | 0.84 | Zaklan 2002 |
| *Hapalogaster dentata* | 0.97 | 0.9 | 1.04 | Goshima et al. 1995 |
| *Oedignathus inermis* | 1.175 | 1.16 | 1.19 | Zaklan 2002 |

**References**

Abelló P, Macpherson E (1991) Distribution patterns and migration of *Lithodes ferox* Filhol (Anomura: Lithodidae) off Namibia. Journal of Crustacean Biology 11(2):261–268

Arnaud PM, Do-Chi T (1979) Résultats préliminaries obtenus sur les lithodes aux iles Crozet, Marion et Prince Edward, pendant la campagne océanographique MD.08. C.N.F.R.A. (Comité national français de Recherche antarctique) 44:135–136

Eldredge LG (1976) Two new species of lithodid crabs from Guam. Micronesica 12:309–315

Faxon W (1893) Reports on the dredging operations off the west coast of Central America to the Galapagos by the U.S. Fish Commission steamer Albatross VI. Preliminary descriptions of new species. Bulletin of the Museum of Comparative Zoology, Harvard College 24:149–220

Goshima S, Ito K, Wada S, Shimizu M, Nakao S (1995) Reproductive biology of the stone crab *Hapalogaster dentata*. Crustacea Research 24:8–18

Guzmán ML, Campodónico I (1972) Fecundidad de la centolla *Lithodes antarctica* Jacquinot (Crustacea, Decapoda, Anomura, Lithodidae), Anales Instituto de la Patagonia 3:249–258

Hiramoto K, Sato S (1970) Biological and **fi**sheries survey on an Anomuran crab, *Lithodes aequispina* Benedict, off Boso Peninsula and Sagami Bay, central Japan (in Japanese with English abstract). Japanese Journal of Ecology 20:165–170

Jewett SC, Sloan NA, Somerton DA (1985) Size at sexual maturity and fecundity of fjord-dwelling golden king crab *Lithodes aequispina* Benedict from northern British Columbia. Journal of Crustacean Biology 5(3):377–385

Lovrich GA, Vinuesa JH (1993) Reproductive biology of the false southern king crab (*Paralomis granulosa*, Lithodidae) in the Beagle Channel, Argentina. Fishery Bulletin 91(4):664–675

MacDonald JD, Pike RB, Williamson DI (1957) Larvae of the British species of *Diogenes*, *Pagurus*, *Anapagurus* and *Lithodes* (Crustacea, Decapoda). Proceedings of the Zoological Society of London 128:209–257

Marukawa H (1933) Taraba-gani chosa [Biological and fishery research on the Japanese king crab *Paralithodes camtschatica* (Tilesius)]. Sui Shi Ho, Tokyo 4(37), pp. 152

Otto RS (1993) Plots of South Georgia island crab data. CCAMLR Workshop on the Management of the Antarctic Crab Fishery. Document WS-Crab-93/94

Somerton DA (1985) The disjunct distribution of the blue king crab, *Paralithodes platypus*, in Alaska: some hypotheses. Proceedings of the International King Crab Symposium Anchorage, Alaska*,* pp 13–14

Somerton DA, MacIntosh RA (1985) Reproductive biology of the female blue king crab *Paralithodes platypus* near the Pribilof Islands, Alaska. Journal of Crustacean Biology 5:365–376

Vinuesa JH (1987) Embryonary development of *Lithodes antarcticus* Jacquinot (Crustacea, Decapoda, Lithodidae) developmental stages, growth and mortality. Physis (Buenos Aires) 45:21–29

Zaklan SD (2002) Evolutionary history and phylogeny of the family Lithodidae. PhD Thesis, University of Alberta
